# Potential use of iPSCs for disease modeling, drug screening, and cell-based therapy for Alzheimer’s disease

**DOI:** 10.1186/s11658-023-00504-2

**Published:** 2023-11-30

**Authors:** Hany E. Marei, Muhammad Umar Aslam Khan, Anwarul Hasan

**Affiliations:** 1 Department of Cytology and Histology, Faculty of Veterinary Medicine, Mansoura University, Mansoura, 35116 Egypt; 2 Biomedical Research Center, Qatar University, 2713 Doha, Qatar; 3 Department of Mechanical and Industrial Engineering, College of Engineering, Qatar University, Doha, Qatar

**Keywords:** Alzheimer’s diseases, Induced pluripotent stem cells, iPSCs, Disease modeling, Drug development, Mechanism of diseases, Regenerative medicine, Cell-based therapies

## Abstract

Alzheimer’s disease (AD) is a chronic illness marked by increasing cognitive decline and nervous system deterioration. At this time, there is no known medication that will stop the course of Alzheimer’s disease; instead, most symptoms are treated. Clinical trial failure rates for new drugs remain high, highlighting the urgent need for improved AD modeling for improving understanding of the underlying pathophysiology of disease and improving drug development. The development of induced pluripotent stem cells (iPSCs) has made it possible to model neurological diseases like AD, giving access to an infinite number of patient-derived cells capable of differentiating neuronal fates. This advance will accelerate Alzheimer’s disease research and provide an opportunity to create more accurate patient-specific models of Alzheimer’s disease to support pathophysiological research, drug development, and the potential application of stem cell-based therapeutics. This review article provides a complete summary of research done to date on the potential use of iPSCs from AD patients for disease modeling, drug discovery, and cell-based therapeutics. Current technological developments in AD research including 3D modeling, genome editing, gene therapy for AD, and research on familial (FAD) and sporadic (SAD) forms of the disease are discussed. Finally, we outline the issues that need to be elucidated and future directions for iPSC modeling in AD.

## Overview of Alzheimer’s disease

Alzheimer’s disease (AD), a fatal condition, is a neurological disease characterized by progressively declining cognitive processes, such as memory and learning, and irreversible neurodegeneration [1]. According to van der Flier and Scheltens [2], AD is a major factor causing dementia, a clinical condition with pathological deterioration of multiple cognitive processes including cognition, language, and behavior. Over 46 million people worldwide suffer from dementia, and at least half of them have Alzheimer’s disease. This number is expected to rise as the average lifespan rises. As the average age of the population rises, AD will have a significant negative impact on individuals, families, and healthcare systems. Despite multiple investigations, identifying the underlying causes and possible treatments for Alzheimer’s disease remains important. All treatments for AD focus on symptom relief and increased quality of life [3].

**Fig. 1:**
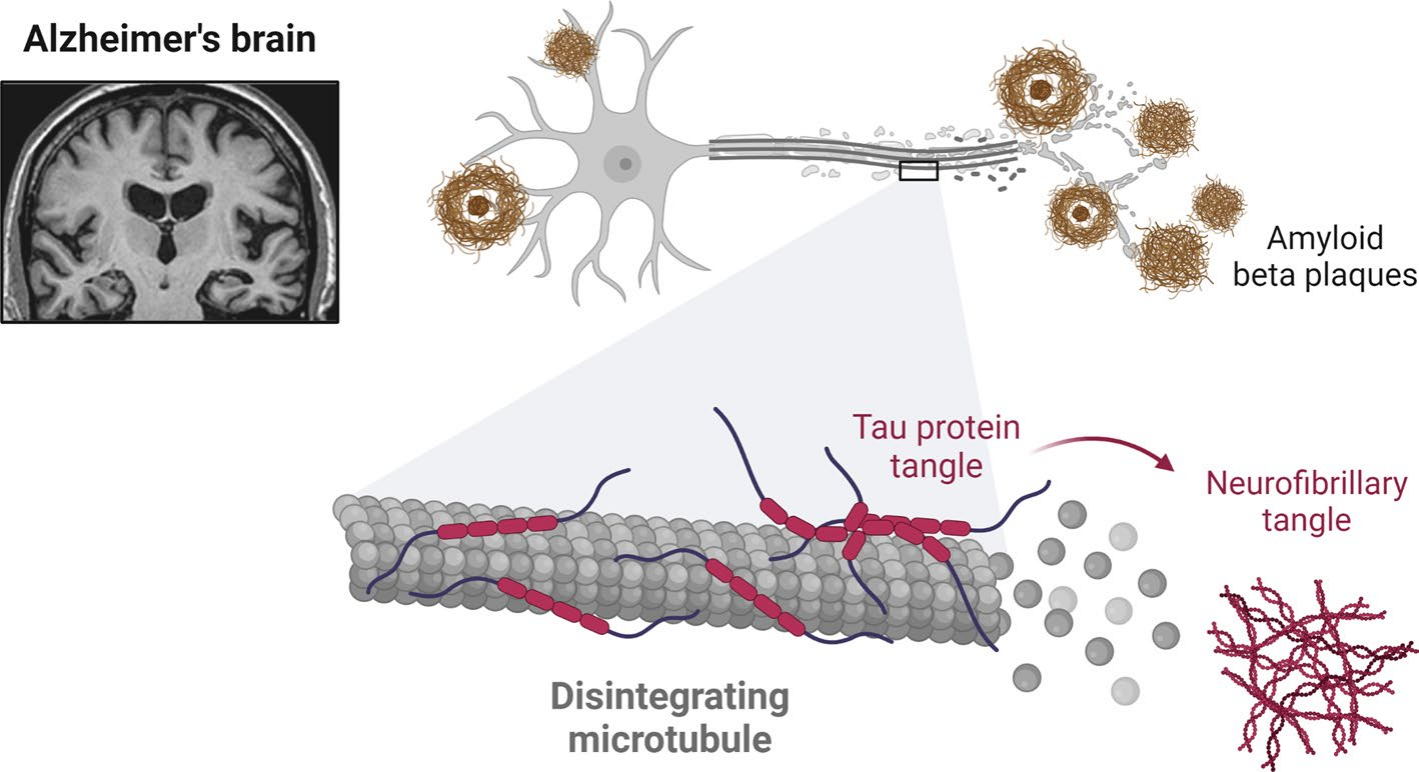
Alzheimer’s Brain (Disintegrating Microtubule). The transmembrane amyloid precursor protein is cleaved by proteases to produce the β-amyloid (Aβ) peptides that make up the majority of the deposits that make up amyloid plaques (APP). The bulk of NFTs are composed of aggregates of hyperphosphorylated tau protein

According to Stefani and Dobson [4], AD is a heterogeneous disorder with two distinct neuropathological features. Development of intracellular neurofibrillary tangles (NFTs) and extracellular amyloid plaques. According to O’Brien and Wong [5], amyloid plaque deposits composed primarily of amyloid beta (Aβ) peptides are generated by proteolytic cleavage of the transmembrane amyloid precursor protein, primarily in neurons (*APP*). Aggregates of hyperphosphorylated tau protein constitute the majority of NFTs [6] (Fig. 1). NFTs and amyloid plaques were first identified more than 110 years ago, but their link to the cause of AD is still not understood [7]. First proposed in 1984, the amyloid cascade theory [8] is supported by extensive preclinical and clinical studies. We convincingly link Aβ to the pathophysiology of AD. In mice models with AD mutations, human genetic investigations have successfully recapitulated age-related neurodegenerative elements of AD, delivering helpful molecular insights into cell-type-specific pathways of AD pathogenesis [9]. The difficulty to apply the findings from rodent studies to clinical trials involving AD patients highlights the requirement for more effective models [10]. Humans and rodents have distinctly different expressions and regulations of a number of key AD-associated proteins, which may have adversely affected the results [11, 12].

Recent studies highlight the importance of using human cells to model human neurodegenerative diseases such as brain cells from iPSCs [9]. Research on modeling patient cells has increased dramatically since Takahashi and Yamanaka’s discovery of induced pluripotent stem cells (iPSCs) in 2007 [1, 13]. These human iPSCs can successfully differentiate into a variety of different cell types, including cortical neurons [14, 15], astrocytes [16–19], and oligodendrocytes [20]. Modeling of patient cells in vitro is not possible due to the limitations of embryo-generated stem cells. Standard methods of mimicking neurodegenerative diseases in iPSCs include taking patient samples (often skin fibroblasts or polymorph nuclear cells (PMNCs) from whole blood) and reprogramming them according to one of several methods [21]. Then, these cells are differentiated into a neurological fate and used as tools to study cellular pathology or to find and test potential therapeutics. More effectively extrapolating preclinical results from a range of neuropsychiatric and neurodegenerative disorders to relevant human populations is possible. This review emphasises how iPSC technology, which is quickly developing, may be used to model AD. In order to gain molecular insights into AD pathogenesis, we also use iPSC-derived brain cell types. This highlights the potential possibility of utilizing iPSCs technology for better translational investigations, such as AD modelling, drug discovery, and cell-based therapy.

### Pathophysiology of AD

**Fig. 2:**
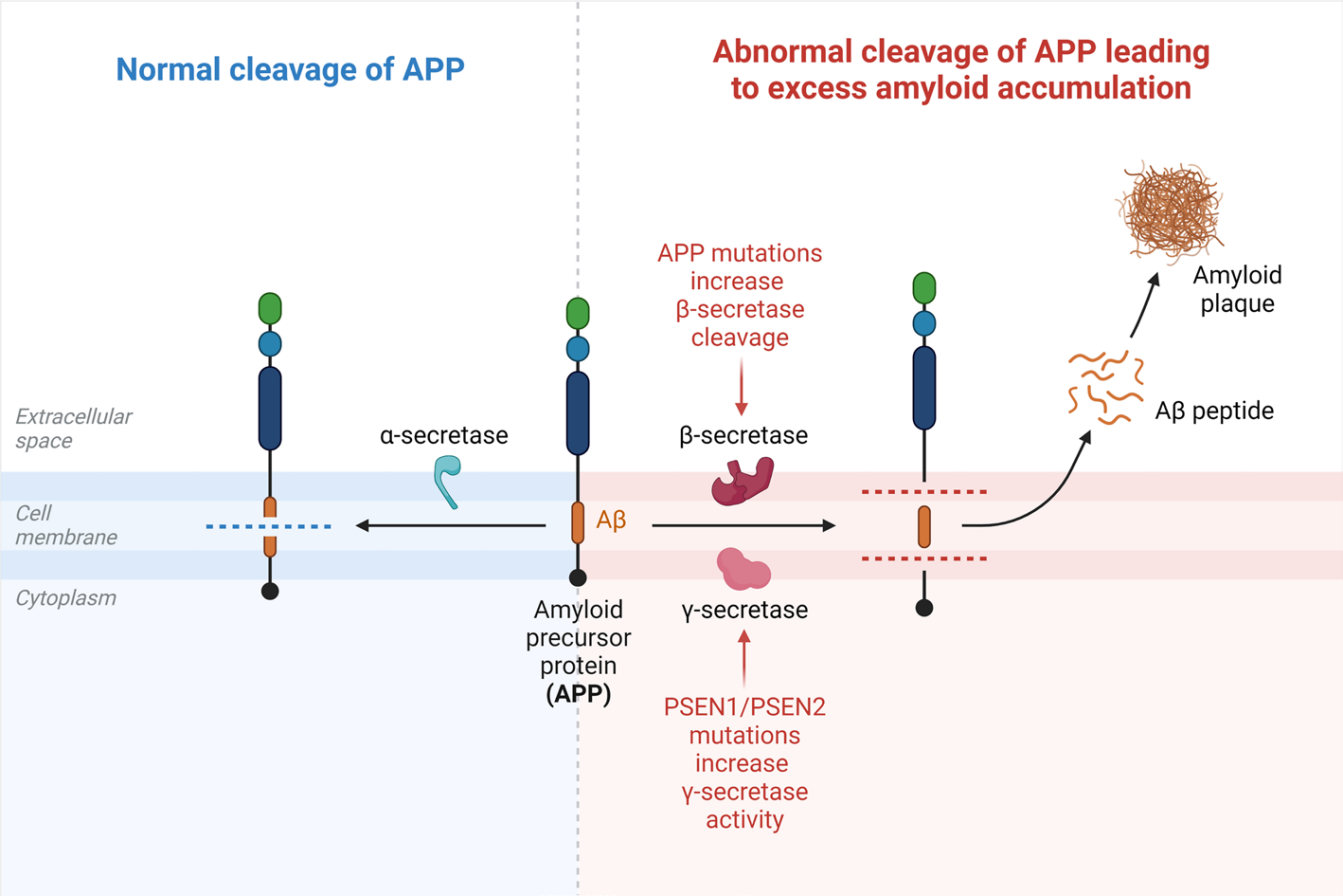
Cleavage of amyloid precursor protein (APP). Aβ-peptides, which are sequentially released from the amyloid precursor protein (APP) by β- and γ-secretase, are responsible for amyloid plaques. In addition, α-secretase and γ-secretase can sequentially cleave APP, yielding non-amyloidogenic fragments. Since APP and β-secretase are primarily expressed in neurons, most Aβ is produced in neurons

Along with the formation of extracellular amyloid plaques and intracellular neurofibrillary tangles containing hyperphosphorylated tau, the pathological indicators of AD also include widespread gliosis, synaptic dysfunction, and neuronal cell death (p-tau) [22]. According to Chow et al. [23] and Bernabeu-Zornoza et al. [24] Aβ peptides sequentially released from the amyloid precursor protein (*APP*) by β-secretase and γ-secretase form amyloid plaques. α-Secretase and γ-secretase can also sequentially cleave *APP*, producing non-amyloidogenic fragments [23]. Since *APP* and β-secretase are highly expressed in neurons, most Aβ is produced in neurons [15] (Fig. 2). The most prevalent Aβ42 and Aβ40 isoforms, which are the subject of AD study, are present in all Aβ species. In human AD brains, these isoforms are present in amyloid plaques [25]. In contrast to other forms, Aβ-42 is formed in dense nuclear plaques in the brain parenchyma and has a high fibrosis rate and insolubility. Because it is more soluble, the most common form of Aβ, Aβ40, causes amyloid to accumulate in blood vessel walls and cause cerebral amyloid angiopathy (CAA). Reduced cerebrospinal fluid (CSF) Aβ42/Aβ40 ratio, suggesting reduced CSF-mediated Aβ clearance and increased buildup of amyloid plaques in the brain parenchyma, is a powerful diagnostic for AD [26]. This study shows that soluble Aβ42 oligomers impair glutamatergic neurotransmission, cause synaptic loss and alter synaptic plasticity, and thus are more detrimental to AD patients than the Aβ protein found in amyloid plaques. [27] In addition to Aβ-induced toxicity, numerous investigations have demonstrated the molecular relevance of altered *APP* metabolism and loss of γ-secretase function as contributing to the pathogenesis of AD [28].

Tau pathology in Alzheimer’s disease typically develops after Aβ pathology and may be brought on by Aβ [29]. The MAPT gene produces the microtubule-associated protein tau. Under physiological conditions, tau is essential for microtubule stabilization, regulation of microtubule assembly dynamics, and axonal transport [30]. Six tau isoforms are produced through alternative splicing of the MAPT gene’s exons 2, 3, and 10 [31]. Tau proteins with 0 and 2 nucleotide repeats are generated by splicing exons 2 and 3, and tau proteins with 3 or 4 microtubule-binding domains are expressed by splicing exon 10 (3R or 4R dew) [32]. During the pathogenesis of AD, tau disease spreads like a prion and follows a stereotypical pattern. The integrity of this structure, which first develops in the locus coeruleus of the brainstem, reveals neuropathology and cognitive function in AD patients [33]. Tau disease begins in the locus coeruleus of the entorhinal cortex and later extends to the hippocampus and neocortex [34]. Entorhinal cortical neurons expressing tungsten-1 are known to send toxic tau to hippocampal neurons [35].

**Fig. 3:**
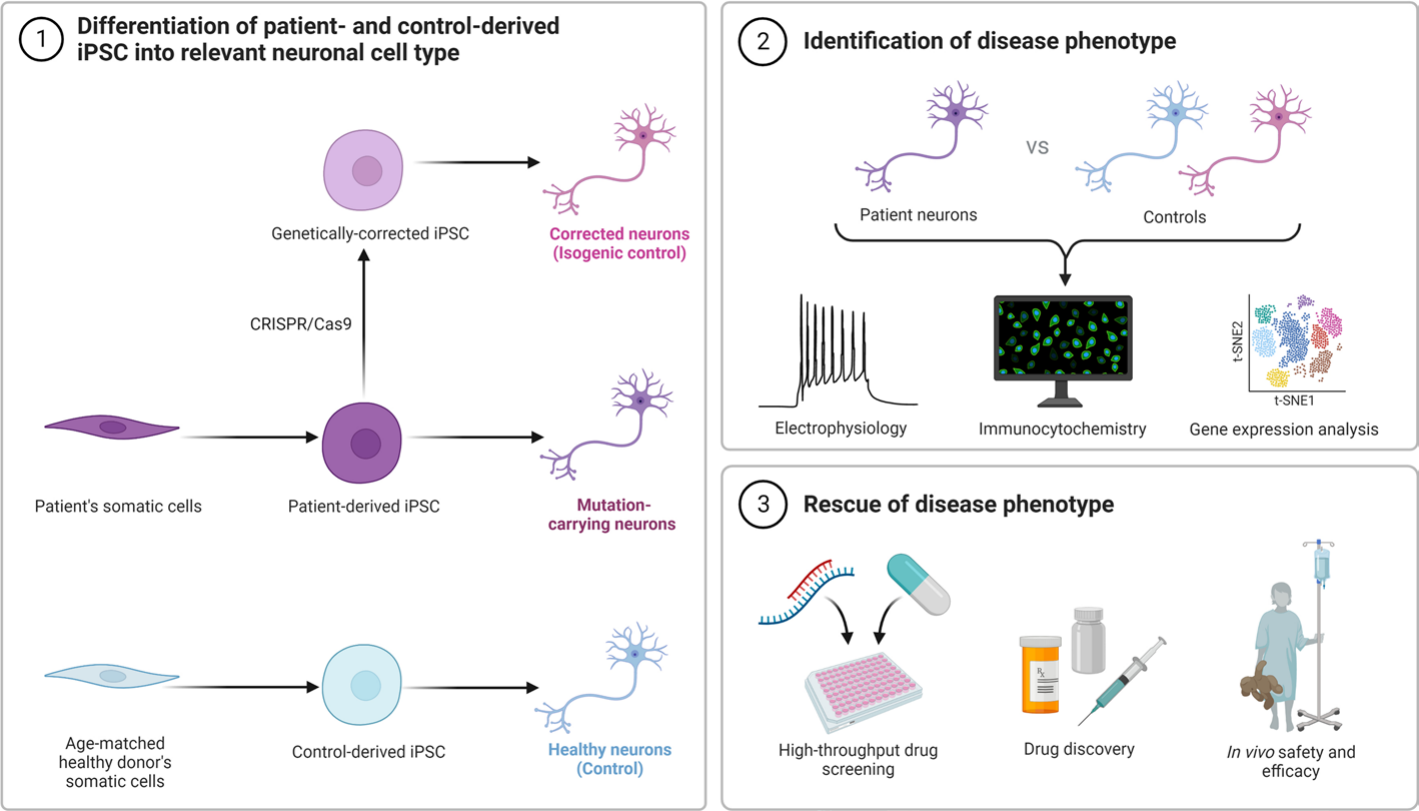
Modeling AD Disease using iPSCs. First, the development of suitable neuronal cell types derived from patient and control iPSC. The next step is to describe the disease phenotype using various functional and genomic analyses. High-throughput drug discovery and screening might be performed on the patient and control iPSC-derived cells

Another important pathogenic aspect of AD is the disturbance of the blood–brain barrier (BBB), and recent studies have shown that degradation of the BBB pericytes contributes to neurovascular dysfunction and exacerbation of Aβ and tau pathology. It has been shown to be related [36]. Interestingly, AD is consistent with the deposition of Aβ, which can signal pericytes to constrict capillaries [37]. There has been a lot of work done to create neurons from adult human brain pericytes that can be utilized to treat AD [38]. Using patient- and control-specific iPSCs for disease modeling has been shown to be beneficial for disease modeling, drug screening, and cell-based therapeutics (Fig. 3). Early-onset familial AD (FAD) and sporadic AD (SAD) are the two main types of AD [39]. The *APP* gene and the *PSEN1* and *PSEN2* genes, which encode presenilins 1 and 2, respectively, are two examples of genes involved in Aβ synthesis that can be mutated and cause FAD [40]. The *APP* and *PSEN2* loci, respectively, contain roughly 30 and 20 recognised changes, and *PSEN1* has been associated with about 200 pathogenic variants [41]. All of these pathogenic FAD gene mutations result in an increase in overall Aβ42 or the Aβ42/Aβ40 ratio [42]. FAD accounts for 1–5% of all AD cases, and most AD cases are sporadic. A genome-wide association study (GWAS) has revealed over 40 genes associated with an increased risk of developing Alzheimer’s disease, including the highly expressed glia-specific genes APOE4, TREM2, ABCA7, and SORL1. GWAS have been used to identify the molecular mechanisms behind AD development [43]. These findings demonstrate that astrocytes and microglia, among other non-cell autonomic neuronal processes, largely contribute to neurodegeneration in AD. According to Holtzmann et al. [39] and Selkoe and Hardy [44], amyloid pathology forms at the onset of both FAD and SAD cases, followed by tau pathology and cognitive impairment.

According to Takahashi and Yamanaka [13], mouse and human fibroblasts can become pluripotent when four different exogenous transcription factors (Oct4, Sox2, cMyc, and Klf4) are overexpressed. At this stage, cells can develop into all kinds of somatic cells. To this point, most iPSC studies have focused on developing cell lines with FAD-associated mutations, as the monogenic pathogenesis of FAD makes it an interesting alternative to model AD from patient-derived cells. It is not unexpected that all induced pluripotent stem cell (iPSC) models of familial Alzheimer’s disease (FAD) have been created by introducing mutations in either the *APP*, *PSEN1*, or *PSEN2* genes. Generally, mutations have the effect of augmenting the production of Aβ, enhancing its tendency to aggregate, facilitating the creation of harmful aggregation structures, and influencing processing to encourage the generation of Aβ42, which is the primary isoform of Aβ implicated in the pathogenesis of Alzheimer’s disease [45]. These genetic changes also result in reduced functionality of γ-secretase. The existence of these genetic modifications has been suggested as supplementary paths to neurodegeneration and Alzheimer’s disease (AD), which have not been extensively investigated [46]. Pathogenic mutations in PSEN1 and *PSEN2* disrupt the catalytic subunit of γ-secretase., which also increase the Aβ42/Aβ40 ratio.

In addition, amyloid plaques and NFTs are produced by trisomy of chromosome 21 which causes Down’s syndrome (DS). This is most likely the result of increased gene dosage, as chromosome 21 contains the gene for *APP*. Overall, due to its monogenic nature, FAD is a perfect illness to simulate in patient-derived iPSCs and provides a well-defined and controllable etiology for the observed pathology [47].

### iPSCs and AD modeling

According to Yagi et al., both secretion and the Aβ42/Aβ40 ratio were increased in developing neurons in *PSEN1* and *PSEN2* mutant FAD [48]. Israel et al. shortly thereafter described the generation of FAD iPSCs in which differentiated neurons from patients with both SAD and *APP* duplication displayed increased phosphorylated tau [49]. Aβ42/Aβ40 ratio and total and phosphorylated tau generally contribute 1.2- to fivefold greater AD pathogenesis, respectively, in subsequent studies using FAD-iPSC-derived models.

Moore et al. generated nerve cells from AD patients with mutations in *PSEN1*, APP, or trisomy 21 and used them to decipher Aβ/p-tau connections in vitro using iPSCs [50]. In this study, they showed a direct correlation between increased levels of total and phosphorylated tau and AD mutations (V717L mutation and *APP* duplication) that increase *APP* dosage. Additionally, they discovered that γ-secretase inhibition (GSI) markedly elevated total tau, whereas γ-secretase modulator (GSM), a substance that specifically disrupts γ-secretase *APP* processing activity, increased total tau. Li et al. discovered that DS neurons displayed a notable increase in the protein p44 [51]. A p53 tumour suppressor protein variant, called p44 has been discovered to produce cognitive deterioration similar to that of late aging and increased tau phosphorylation in mouse models when overexpressed [52].

Although replication of neurodegenerative changes in iPSC-derived nerve cells can be challenging, recent studies have demonstrated that there is a significant gene expression overlap and link between Aβ and tau species. It has demonstrated the value of using iPSCs to clarify the fundamental pathophysiology of AD in humans [53]. Another team demonstrated that neurons with *APP* and *PSEN1* mutations exhibited reduced general autophagy and lysosomal activity by blocking γ-secretase with γ-secretase inhibitors (GSI), and found that FAD mutations have further suggested that is a direct cause of autophagy impairment [54]. It is important to note that healthy neurons had mitochondrial dysfunction when extracellular vesicles from individuals with *PSEN1* mutations exhibited high Aβ42/Aβ40 ratios. Furthermore, lysosomal dysfunction caused by impaired autophagy resulted in increased pathogenic extracellular vesicles with high Aβ42/Aβ40 ratios [55].

iPSCs are an excellent cell source for studying pathogenic changes in human neurons associated with AD. Early studies showed that some of the key regulators and *APP* processing machinery were expressed in human iPSC-derived neurons, including β-secretase and γ-secretase, and a range of different *APP* and Aβ37-42 isoforms, it has been shown to be expressed at the N-terminus truncated Aβ2-40 [56]. Additionally, in human iPSC-derived neurons, many tau isoforms, including 3R and 4R tau, display a developmental pattern [57]. In cortical neurons created from human iPSCs and mouse models of tauopathy, increased neuronal activity promotes the distribution of tau and favors the development of tau disease [58]. Low-density lipoprotein receptor-related protein 1 (LRP1) has recently been identified as a receptor that controls the endocytosis and spread of tau, as shown in human iPSC-derived neurons [59]. Human iPSC-derived cortical neurons and organoids with FAD mutations in *PSEN1* (PS1-DE9 and M146V mutations) and *APP* (KM670/671NL; Swedish mutations) exhibit abnormally increased electrical activity when compared to their isogenic WT controls. [60]. HiPSCs were generated using dermal fibroblasts from AD patients harboring the *PSEN2* N141I missense mutation. The N141I missense mutation was corrected through the utilization of genome-editing technologies, resulting in the identification of iPSC colonies that exhibited recognition by pluripotent marker labelling [61].

We have gained a better understanding of the molecular mechanisms behind AD pathogenesis through the characterization of iPSC-derived neurons with FAD mutations. In animal models of AD, there is mounting evidence that Aβ causes aberrant tau production and accumulation [62], and this pathogenic characteristic may be reproduced in neurons made from iPSCs. The *APP* London mutation (V717I) causes aberrant *APP* cleavage and enhanced Aβ production in forebrain neurons made from iPSCs, which raises levels of total tau and p-tau [63]. These results demonstrate that tau pathology is an unfavourable effect of Aβ and that treating Aβ early in the course of AD development may be a successful therapeutic strategy. Research on iPSC-derived neural progenitor cells (NPCs) and neurons with FAD or SAD mutations/mutations, including hers, has shown that her FAD mutations in the *APP*, *PSEN1*, *PSEN2*, and APOE (APOE4) loci increase levels of Aβ-induced p-tau in wild-type (WT) neurons [64–66].

**Fig. 4:**
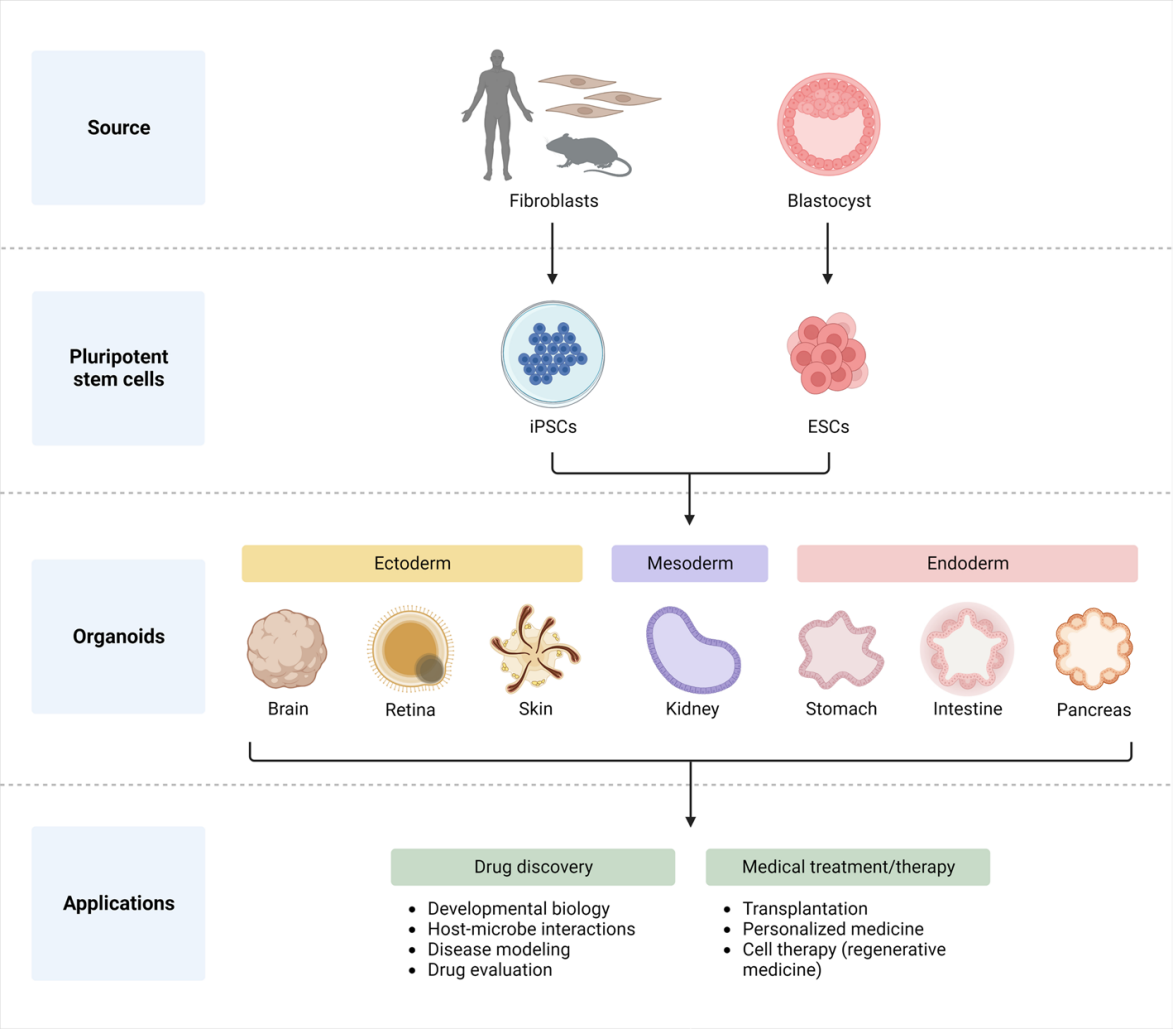
Organoid Generation from Pluripotent Stem Cells. Blastocysts or somatic cells can be used to create pluripotent stem cells like iPSCs and ESCs. These cells are differentiated into cell types that are embryologically separate and could be used to create organ-specific organoids. The application of the organoids for cell therapy, transplantation, customised medicine, and drug discovery follows

The importance of a 3D environment in recreating important AD clinical characteristics is highlighted by the observation that human neural progenitor cells overexpressing FAD *APP* and *PSEN1* mutations have an increased Aβ42/Aβ40 ratio that encourages the production of neurofibrillary tangles in a 3D culture system [67, 68]. Furthermore, the development of brain organoids, which have served as representations for AD, can produce a 3D environment. (Fig. 4). Using microglia made from iPSCs, several research studies have established a viable disease modeling method. However, there are considerable technological constraints to using these human microglia. For instance, it is challenging to study interactions between various brain cell types and microglia in a controlled culture environment, and alterations in microglia transcriptome are sensitive to medium composition [69]. iPSC-derived microglia are relevant for study into AD, according to recent studies [70, 71]. However, because microglia and neurons have different embryonic origins, it can be challenging to discriminate between the two. Early on in the process of hematopoiesis, progenitors found in the yolk sac give rise to microglia, which are later produced by mesoderm that migrates to the neural tube [72]. Thus, microglial cells have a separate embryonic origin from neurons, astrocytes, and oligodendrocytes, which are formed from neuroectoderm and can be isolated from NPCs [73]. In order to increase human iPSC-derived microglia through lineage status analogous to hematopoietic progenitor cells (HPCs) in vitro, numerous approaches have been devised that provide key components for imitating microglial embryonic development [19, 74].

iPSC-derived microglia have also been used to explore the signaling processes of AD-related genes. For instance, AD is prevented by the PLCG2 functional gain-of-function variation P522R [75]. Recently, iPSC-derived TREM2- and PLCG2-deficient microglia were found to have similar clinical features, including increased lipogenesis, impaired phagocytosis, and decreased cell viability [76]. This study’s use of genetically modified iPSC-derived microglia supported this finding by demonstrating that PLCG2 was required for downstream TREM2 signaling [76]. These iPSC-based research findings demonstrate that intrinsic microglial dysfunction and AD are related. Single-cell RNA-sequencing (scRNA-seq) studies revealed that transplanted iPSC-derived microglia maintained their identity and had a range of gene expression patterns that were strikingly similar to those of primary human microglia [77]. An equivalent model of microglial transplantation using human embryonic stem cells has also been reported [78]. Microglia derived from TREM2-deficient human iPSCs replicated key clinical features of TREM2-deficient human AD brains. These include defects in APOE phagocytosis and failure to surround amyloid plaques [79]. Furthermore, scRNA-seq studies revealed that transplanted TREM2-deficient microglia failed to upregulate the human DAM gene. In a previous study, similar conclusions about the decreased function of TREM2 were reached [12]. In a different study, human iPSC-derived microglia from people with the TREM2 R47H mutation were implanted into neonatal mouse brains. This experiment showed decreased susceptibility to amyloid plaques and decreased lipid droplet formation [80], further highlighting TREM2’s significance in the setting of AD. Together, these investigations indicate promise for disease modeling approaches using iPSC-derived microglia. Human iPSCs could be used to differentiate oligodendrocytes, and these cells were incorporated into brain organoids and successfully survived after being transplanted into the brains of myelin basic protein-deficient mice [81, 82]. Currently, the iPSC model of AD oligodendrocytes to study oligodendrocyte function during AD pathogenesis is not published. Astrocytes can be distinguished from human iPSCs and have been used to study disease processes associated with AD [9, 19]. According to several studies [74, 83], atrophy, increased Aβ secretion, altered inflammatory responses, aberrant calcium signaling, increased oxidative stress, and neural support are all linked to iPSC-derived astrocytes with *PSEN1* mutations. The morphology of astrocytes from APOE4-positive SAD patients also changed, leading to an increase in the production of inflammatory cytokines, a decrease in the absorption of Aβ, a breakdown of lipid homeostasis, and an accumulation of lipid droplets [64, 84]. TNF-α released from microglia is capable of activating iPSC-derived astrocytes and interacts with microglia via complement C3 [19]. In addition, astrocytes secrete interleukin-3 (IL-3), which draws microglia and activates them to eliminate Aβ and tau in response to stimuli associated with AD [19, 85]. Oligodendrocytes can be differentiated from human iPSCs, and these cells have been integrated into brain organoids and successfully survived after being injected into mouse brains lacking myelin basic protein [1, 2]. To investigate the role of oligodendrocytes during AD pathogenesis, there are currently no published iPSC models of AD oligodendrocytes available.

Human induced pluripotent stem (hiPS) cells underwent a process of cellular differentiation, resulting in the emergence of neuronal cells that exhibited the expression of the forebrain marker, Foxg1, as well as the neocortical markers, Cux1, Satb2, Ctip2, and Tbr1. The neuronal cells produced from induced pluripotent stem cells (iPSCs) also exhibited the expression of amyloid precursor protein, β-secretase, and γ-secretase components. Furthermore, these cells demonstrated the ability to secrete Aβ into the conditioned media. The generation of Aβ was hindered by the administration of a β-secretase inhibitor, a γ-secretase inhibitor (GSI), and a non-steroidal anti-inflammatory drug (NSAID). However, notable variations in the response to these three treatments were observed between the early and late stages of differentiation. During the first phase of differentiation, the administration of GSI therapy resulted in a rapid rise in Aβ levels at lower doses (referred to as Aβ surge), followed by a significant decrease in Aβ production. The findings of this study suggest that the neuronal cells obtained from human induced pluripotent stem cells (hiPS cells) display functional β- and γ-secretases, which are known to be involved in the generation of Aβ. However, it is important to note that in order to effectively screen anti-Aβ drugs utilising these hiPS cell-derived neuronal cells, it is necessary to ensure an adequate level of neuronal development [86].

Wang et al. (2017) devised a resilient high-content screening assay for the purpose of identifying compounds that have the ability to lower tau levels. In their study, they specifically focused on the Library of Pharmacologically Active Compounds (LOPAC) and successfully discovered adrenergic receptor agonists as a distinct class of compounds that exhibit the capability to decrease endogenous human tau. These methodologies facilitate the utilisation of human neurons for conducting high-throughput screenings of pharmaceutical compounds aimed at addressing neurodegenerative disorders [87]. In their study, Kondo et al. employed human-induced pluripotent stem cell (iPSC)-derived neurons, which possess the unique characteristic of human-specific drug responsiveness, in order to facilitate medication development targeted towards Alzheimer’s disease (AD). Through the utilisation of induced pluripotent stem cell (iPSC)-based screening of pharmaceutical compounds and employing chemical clustering techniques, the researchers were able to identify a specific combination of pre-existing medications that exhibited a synergistic effect in enhancing the phenotypes associated with (Aβ) accumulation in cells affected by Alzheimer’s disease (AD) [88]. In order to gain insights into the genetic basis of Alzheimer’s disease (AD), Kondo et al. (2023) successfully created models of AD using patient-derived cells to with the aim to provide a deeper understanding of the genetic factors that contribute to sporadic Alzheimer’s disease (SAD) cases [89].

### iPSCs and drug screening for AD

**Fig. 5:**
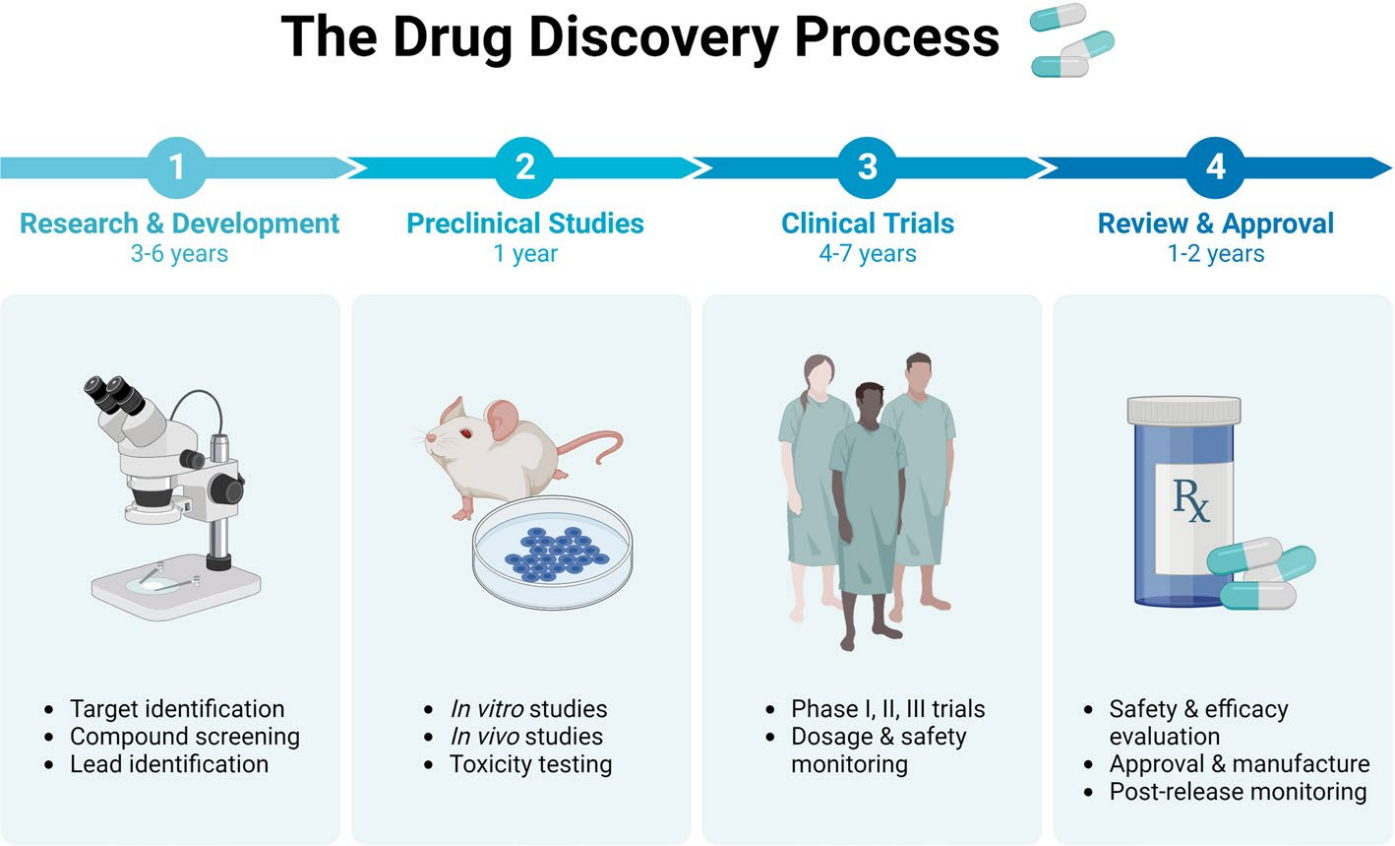
The Drug Discovery Process. The drug discovery process involves the identification of different therapeutic targets based on extensive functional and genomic studies. Drugs designed to target different targets are tested in vitro, in vivo and toxicity testing to provide convincing preclinical evidence. Provision of preclinical evidence will qualify candidate drug for clinical trials followed by review and approval after evaluation of safety and efficacy

As part of the drug development process, various therapeutic targets are identified through intensive functional and genomic research. Drugs developed for different targets are examined through in vitro, in vivo and toxicology studies to obtain meaningful preclinical data. Drug candidates qualify for clinical trials by providing preclinical evidence that is reviewed and approved after safety and efficacy assessments (Fig. 5). iPSC isolated from AD patient are undoubtedly a powerful platform for identifying new drugs and interesting targets, nonetheless, their acceptability and safety to people are often unpredictable [47]. As iPSC-derived CNS cell types offer novel AD therapies, it remains to be seen whether they outperform current preclinical models in terms of translational efficacy. The field of drug development holds significant scientific significance in relation to the iPSC FAD model. The aforementioned investigations have demonstrated that GSI contributes to the understanding of the physiological mechanisms behind Alzheimer’s disease (AD). It is noteworthy that GSI has been extensively examined in the context of generating and evaluating AD-induced pluripotent stem cells (iPSCs) [50, 82].

The ability of iPSC lines to respond to potential pharmacological therapies can be assessed based on the mechanism of GSI preventing Aβ production [48, 54]. In addition, the therapeutic potential of GSIs, especially the latest second-generation GSIs, has been investigated using iPSCs [90]. Although the in vitro results of screening GSMs for therapeutic potential with iPSCs were encouraging [48, 54, 90], the success of subsequent studies was limited by weak drug-like properties [91]. Using patient-derived iPSCs, drugs that do not manipulate γ-secretase have also been tested with some success [92]. Tau in *APP* early mutant neurons was successfully reduced by Aβ antibodies as seen by Muratore et al. [63]. The small molecule N-butylidenephthalide, which is derived from chloroform extracts of Angelica sinensis, reduces total tau and phosphorylated tau levels in DS neurons, but neither Aβ-42 nor the Aβ-42/Aβ-40 ratio show any discernible decreases [93]. Additionally, when given to both FAD and SAD neurons, the natural polyphenol apigenin, which is present in many plants, demonstrated neuroprotective properties against inflammatory stress brought on by microglia [94]. Cholesterol metabolism has also been discovered as a possible druggable target for FAD, as *APP*-FAD mutations result in elevated cholesterol esterase, which has been shown to affect both Aβ and tau [95].

Drug testing of patient-derived SAD-iPSC models is an important research area as SAD accounts for more than 99% of all AD cases [96–98]. Similar to FAD, GSI has been investigated as a validator for drug screening in SAD neurons. Another example of the diversity of pathophysiology identified in SAD cell lines come from Hossini et al., they performed GSI on two of his SAD cell lines and found reduced phosphorylated tau in only one of them [99]. Israel et al. demonstrated that phosphorylated tau and GSK-3 activity were decreased by γ-secretase inhibitors but not by GSI. This was a common feature of FAD derived cells [49].

### iPSCs and genome editing for AD

AD is still untreated with effective targeted therapy, which is one of the causes of a significant public health burden. Genome engineering and induced pluripotent stem cells (iPSCs) are two revolutionary technologies being developed simultaneously that could change this. Investigating the underlying causes of disease and identifying therapeutic targets in AD is hampered by the largely inaccessible human central nervous system. Heterogeneous in vitro cell cultures and animal models shed light on the pathophysiological mechanisms underlying various neurological diseases, including AD. However, these models only partially reconstruct disease development and do not accurately reflect human physiology, metabolism, or homeostasis [100]. As a result, failure rates are high in both innovative therapeutics discovery and clinical trials for neurological disorders. Thus, iPSC patient-derived neurons provide a unique in vitro model for studying AD. They provide a limitless supply of genetically identical patient-derived cells that enable the study of disease-associated signaling pathways. They offer humanized models for testing new medicines, which might hasten their adoption. Additionally, they offer a trustworthy source of cells for cell replacement therapy in neurological conditions like AD. Since its introduction, gene-editing techniques have proven useful in creating in vitro disease models [103–107].

The discovery of the DNA-binding zinc finger nuclease (ZFN) technique boosted the effectiveness of genome editing in mammalian cells [108], which led to the creation of the first knock-out rats [108]. Patient-derived iPSCs have been used to correct genetic mutations using ZFN-based genome editing [109] or to incorporate known disease-associated mutations into iPSCs produced from healthy people [110]. With the discovery of transcription activator-like effector nucleases (TALENs), which have shown to be valuable tools for the creation of animal models, genome editing technology was further refined [111]. TALENs have also been applied in neuropathy research by introducing disease-causing mutations into control iPSCs and/or reversing genetic mutations in patient-generated iPSCs [112]. This has increased confidence in the development of underlying mechanisms and therapeutic strategies.

Clustered regularly interspaced short palindromic repeats (CRISPR) and CRISPR-associated protein (Cas9) technologies quickly developed after TALENs technology and have been shown to be capable of editing the mammalian cell genome in both culture and animal models [113]. Compared to ZFNs or TALENs, CRISPRCas9 uses different DNA cleavage and binding modules. But in order to specifically bind target DNA sequences and activate Cas9, the CRISPR-Cas9 system depends on CRISPR RNA (crRNA), trans-activating RNA (transRNA), and a particular natural endonuclease. CRISPR-based gene editing technique has demonstrated effectiveness for gene alteration, gene expression regulation, epigenetic regulation, and chromatin manipulation at both the single-gene level and large-scale screening due to its versatility and robustness [114]. Because of this, CRISPR-based technology has quickly taken over as the go-to technique for altering genomes, particularly in iPSC model systems. Furthermore, genome editing of control strains allows multiple variants to be studied simultaneously in the same genetic context. This may be more practical than assembling a substantial number of patient strains. In order to explore related disease pathways [115].

Another study that corrected LRRK2 mutations revealed both LRRK2-dependent and LRRK2-independent effects that are probably genetically influenced and connected to different familial Parkinson’s disease clinical presentations. It presents characteristics and varying degrees of severity [116]. Isogenic regulation can also indicate that some cell phenotypes depend on the genetic background even under monogenic conditions.

Different apolipoprotein E4 gene genotypes are associated with the risk of sporadic Alzheimer’s disease (APOE4) [117]. Several studies, a patient’s APOE4 gene was converted to APOE3 by iPSCs, while a neutral-risk (APOE3) gene was converted to APOE4 (high risk) by healthy individuals. This ‘rescue’ of iPSC risk status from individuals prone to develop AD later in life impairs the inability of glial cells to clear extracellular Aβ and increases Aβ aggregates in cerebral organoids [118].

Understanding the pathophysiological pathways associated with disease-related gene alterations has been enabled by gene editing in iPSC systems. However, genome engineering can be combined with transcriptome studies to more thoroughly investigate the underlying causes of disease. In order to research AD in early-onset Down syndrome patients, CRISPR-Cas9 was utilized to remove the extra copy of *APP* from the T21 lineage, and inducible CRISPRa was employed to boost *APP* gene expression [119]. Levels of the *APP* gene have been found to be associated with Aβ formation, but not with other cellular traits associated with AD such as apoptosis. The use of CRISPR screens to uncover disease pathways is discussed in greater detail below. In AD, certain brain areas seem to be particularly impacted by the development of Aβ plaques. Brain areas in AD imply vulnerability, and neurons from patient-derived iPSCs carrying *APP* mutations were differentiated with either a caudal (hindbrain) or rostral (forebrain) destiny. Forebrain neurons displayed a more severe tau reaction [120]. His study of the effect of APOE4 genotype in microglia [74] shows that SAD is more likely to affect some cell types than familial AD. Utilizing the pluripotency of iPSCs could help identify potential illness causes and tissue-specific treatment options. Oikarie et al. investigated the impact of familial AD mutations in *PSEN1* on the development of the blood–brain barrier (BBB) by generating induced brain endothelial cells (iBECs) from patient-derived and isogenic lines [121]. Mutant iBECs showed abnormal expression of adherin and tight junction proteins. This could be a novel way to improve CNS medication delivery in AD because AD and isogenic iBECs responded differently in iBEC cultures.

In a separate study focused on familial AD (*APP*), 200 heterozygous disease-causing mutations in presenilin isoforms (*PSEN1* and *PSEN2*) and amyloid precursor protein were screened using the CRISPR-Cas9 system [111]. Cortical neurons generated from multiple genomically altered iPSC lines were subjected to transcriptomic and translational analyses, which revealed that AD family mutations in two distinct genes are connected to the endocytic/endosomal trafficking pathways previously linked to late-onset AD. It turned out to have overlapping effects. By demonstrating that the genesis of familial and sporadic AD may share a network of cellular and molecular changes, our finding offers a shared therapeutic objective. In light of this, combining CRISPR KO and CRISPR KI screening methods with iPSC-based illness modelling may enhance our comprehension of pathophysiological signaling networks and direct therapeutic strategies for neurological diseases.

As indicated earlier, new developments in electrophysiology and transcriptome analysis have demonstrated that, even after prolonged culture, iPSC-derived neurons only represent late stages of foetal development [122, 123]. This is acceptable for early-onset and/or highly penetrant monogenic disorders with cell-autonomous phenotypes, but it is challenging to identify in vitro late-onset phenotypes or those in which environmental variables play a significant role. There are still worries that it won’t be accurately duplicated. Progesterone, telomere shortening, expression, direct differentiation, pharmacological signaling, and other mechanisms can inhibit this when reprogramming has not taken place [124]. A number of factors, such as reprogramming-induced epigenetic alterations and genomic instability, background genetic dispersion, and variations in differentiation propensity, contribute to the innate diversity and heterogeneity of iPSC-derived neurons [125].

To study juvenile Alzheimer’s disease in a patient with Down’s syndrome, excess copies of *APP* from T21 strain we removed using CRISPR-Cas9 and *APP* gene expression was boosted using inducible CRISPRa [119, 126]. The use of iPSC-based disease models for both Mendelian and more complicated neurological illnesses has been transformed by genome editing. The enhanced accuracy of CRISPR gene editing, promoter regulation, and epigenome editing, along with an individualized patient-derived iPSC model system, may result in a paradigm change in how neurological illnesses are seen and treated.

### IPSCs-cell based therapy for AD

**Fig. 6:**
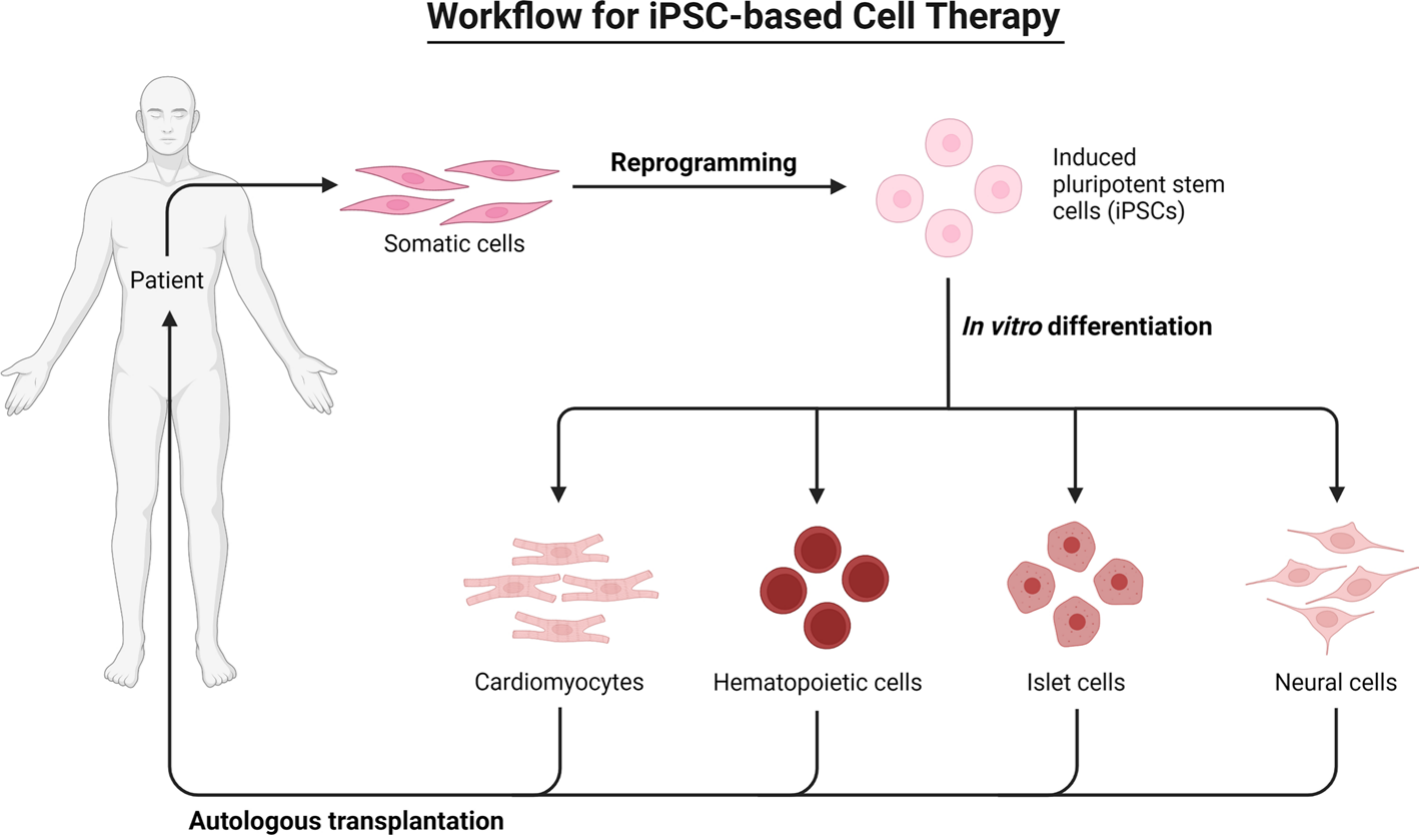
Workflow for iPSC-based Cell Therapy. The somatic cells undergo iPSC reprogramming. Following the differentiation of the iPSCs into various cell types utilising various particular methods, the patient receives an autologous transplant

Since the discovery of iPSCs, innovative techniques utilizing cells produced from iPSCs have revealed crucial insights into the pathogenesis of AD and prospective AD therapies. A number of recent studies using animal models show that cell replacement therapy can help alleviate disease conditions and improve cognitive performance. In the following section, we reviewed the advantages, disadvantages, applicability, and potential use in clinical settings, safety and ethical considerations of cell replacement therapy (Fig. 6).

In clinical studies for AD, disease-modifying treatments have been explored extensively, but nearly all of them were abandoned in phase 3 trials because they either did not show any cognitive benefit or had severe adverse effects. (https://Clinicaltrials.gov). Acuranumab, a human IgG1 antibody, recently received accelerated FDA approval for the treatment of all stages of dementia. But there has been a lot of debate about the FDA’s approval of aducanumab for AD. This is due to the fact that aducanumab was excluded from several clinical trials and did not show cognitive advantages in phase 3 trials [127]. These extracellularly focused strategies may rarely restore all of the damaged neurons. The binding characteristics of lecanemab, aducanumab, and gantenerumab to various Aβ species were investigated using inhibitory ELISA, immunodepletion, and surface plasmon resonance techniques. All three antibodies had a modest affinity for binding monomers. Nevertheless, it should be noted that lecanemab and aducanumab had very low affinity towards monomers, whilst gantenerumab shown a comparatively higher binding affinity. Lecanemab exhibited a notable characteristic in that it demonstrated a binding strength that was ten times greater towards protofibrils as compared to fibrils. Aducanumab and gantenerumab had a higher affinity for binding to fibrils compared to protofibrils [128]. The binding characteristics of lecanemab, aducanumab, and gantenerumab to various Aβ species were investigated using inhibitory ELISA, immunodepletion, and surface plasmon resonance techniques. All three antibodies had a modest affinity for binding monomers. Nevertheless, it should be noted that lecanemab and aducanumab had very low affinity towards monomers, whilst gantenerumab shown a comparatively higher binding affinity. Lecanemab exhibited a notable characteristic in that it demonstrated a binding strength that was ten times greater towards protofibrils as compared to fibrils. Aducanumab and gantenerumab had a higher affinity for binding to fibrils compared to protofibrils. The findings of this research demonstrate distinct binding profiles exhibited by lecanemab, aducanumab, and gantenerumab, which could potentially elucidate the reported clinical outcomes pertaining to the efficacy and adverse effects associated with these antibodies [129]. There is recent evidence that the classical amyloid hypothesis might not fully reflect all aspects of AD and that, for example, tau pathology even precedes the formation of plaques. In a way that the tau pathology is still benign and “boosted” by plaque formation [130–132].

Therapies generated from stem cell-based cell replacement to replace missing or defective neurons can alleviate these problems and make them functional. It has sped up the creation of improved stem cell treatments [13].

Since 1995 [133], when mesenchymal stromal cells (MSCs) were initially utilized as therapeutic agents in clinical trials, cellular therapy has drawn interest from all around the world [134]. MSCs have the benefit of being easily accessible from tissue sources and can be generated in great numbers utilizing straightforward culture techniques (135, 136). MSC transplantation in AD animal models has been shown to be both secure and effective, according to a meta-analysis study. MSCs have recently been used extensively in preclinical animal studies of AD to cure or palliate symptoms. Human umbilical cord (hUC-MSCs) were found to target hyperphosphorylated tau and improve synaptic plasticity in a senescence-accelerated mice model of Alzheimer’s disease. Hepatocyte growth factor is secreted to promote structural and functional repair of damaged brain cells (HGF) [137]. MenSCs made from human menstruation have been marketed as a potential AD treatment to lessen the AD pathology in AD model mice [138].

According to a recent study, dental pulp-derived MSC transplantation can improve cognitive function and raise hippocampal neuronal activity, pointing to possible therapeutic uses for Alzheimer’s disease [139]. In a 3xTg-AD animal model, bone marrow-derived MSC transplantation can also lessen the inflammatory response and tau phosphorylation (BM-MSCs) [140]. Notably, a different study demonstrated that, as compared to BM-MSCs, human neural crest-derived nasal turbinate stem cells dramatically enhanced cognitive function and decreased A42 levels in a 5xFAD mouse model [141]. In mouse models of AD, MSC, or MSC-conditioned medium (MSC-CM), transplantation may improve mitochondrial function and reduce mitochondrial oxidative stress, representing a potentially effective therapeutic strategy [142].

Immune reaction may be brought on by MSC transplantation. Extracellular vehicles (EVs) created from MSCs might be a different strategy because they can pass through the BBB and mimic the advantages of MSCs [143]. MSC-derived EVs have been shown to improve cognitive function and reduce AD pathology when transplanted into animal models of disease [144, 145]. According to one study, giving MSC-derived EVs to patients caused a shift in the pro-inflammatory to anti-inflammatory phenotype of macrophages, which may have an impact on immunological responses and neuroprotection [146]. Another study found that MSC-EVs could prevent hippocampal neuronal loss in her AD mice from being exacerbated by her Aβ42-induced synaptic dysfunction [147, 148]. Notably, exosomes from MSCs reduced Aβ production by modulating α- and β-secretase expression and induced neuronal death by elevating miR-223 levels. According to Liu et al. [149], lateral ventricle injection of BMSC-derived exosomes can lessen cognitive impairments in a mouse model of sporadic AD. Restoring the brain’s depleted NSC pool can restore function to a malfunctioning cerebrum, which suggests a viable treatment strategy [150]. One neurodegenerative disease that benefits from the use of multipotent self-renewing cells is AD. They can essentially form the three major cell types of the nervous system: neurons, astrocytes, oligodendrocytes. This is markedly different compared to lineage-specific brain progenitors. The capability of MSCs to differentiate into bona fide, functional neural cells is highly doubted and the up regulation of certain, isolated neuron-specific proteins should not be regarded as successful (trans-) differentiation. While beneficial effects of MSC transplantation have been observed in various diseases, even without generation of functional cell types in disease, these are mostly attributed to supportive effects of the transplanted cells, as correctly cited, by EVs or other trophic factors [149, 150].

Human olfactory bulb (OB)-derived NSCs (OB-NSCs) have previously been shown to have the ability to survive, proliferate, differentiate, and correct cognitive and motor deficits associated with AD and PD rat models, respectively [151–155]. Recently, it has been proposed to use carbon nanotubes (CNTs) to enhance NSC differentiation and survival after in vivo transplantation. In order to test if CNTs may enhance human OBNSCs’ therapeutic potential for treating cognitive impairments and neurodegenerative lesions, we co-engrafted CNTs and human OBNSCs in a rat model of Trimethyltin (TMT) neurodegeneration (TMT-neurodegeneration). According to the results of the current work, TMT-induced rat neurodegeneration model cognitive impairments and neurodegenerative alterations might be reversed by engrafting human OBNSCs-CNTs. Additionally, the engrafted OBNSCs appeared to be supported by the CNTs, boosting their propensity to develop into neurons as opposed to glia cells. The current study’s findings demonstrate that CNTs can significantly boost human OBNSCs’ therapeutic potential, making this novel therapeutic paradigm a possible option for cell-based therapy of numerous neurodegenerative illnesses [156].

According to Zhang et al. [157], hNSC transplantation can improve memory in P301L mice by significantly reducing aberrant tau aggregation by controlling a number of proteins, mostly those involved in neurogenesis and long-term potentiation. It’s interesting to note that intranasal transplantation of hNSCs can improve conditions similar to AD, as well as finally reverse the cognitive impairment of AD model mouse by boosting adult hippocampus neurogenesis. [158]. As an alternative to NSCs, extracellular vesicles can be used since they have antioxidant, anti-inflammatory, and anti-apoptotic capabilities that are similar to those of NSCs [159]. Using EVs obtained from various iPSC-derived brain cell types. You et al. [160] found that astrocyte-specific EV-enriched hub modules may contribute to AD pathology and cognitive decline showed that it is related to Additional studies using NSC-derived EVs demonstrated improvements in cognitive deficits, synaptic activity, mitochondrial function, and inflammatory responses in AD mouse models [161]. Human embryonic stem cells (hESCs) are one of the safest sources of stem cells for transplantation therapy, notwithstanding the ethical issues they brought up. Medial ganglionic eminence (MGE)-like progenitor cells derived from hESCs have the potential to cure neurological diseases, according to Liu et al. [162], when transplanted into AD animal models, iPSCs pretreated with ESC protein extracts have been demonstrated to decrease Aβ plaque development and exacerbate cognitive impairments. Furthermore, transplantation of thymic epithelial progenitor cells (TEPs) generated from *APP*-/- ESCs may provide a new therapeutic option for AD patients. Peripheral delivery of immune and matrix regulatory cells (IMRC) generated from human ESCs as a potential therapy for AD. Peripheral delivery of hESC-derived immune and matrix regulatory cells has also shown promise as a treatment for AD.

By stimulating neuronal development and real-time tracking of NSCs in vivo, encapsulated nanoparticles can be administered into NSCs in animal models to alleviate Aβ deposition and cognitive deficits brought on by neurodegeneration. Notably, 6-month results continue to demonstrate improvements in learning and memory deficiencies [163].

In SAMP8 mice, Daz-Moreno et al. [164] found that intracranial injection of antiaging compounds could prevent hippocampal damage caused by pathological aging. These results may shed light on the problems that stem cell transplantation has in maintaining long-term efficacy. It has been used in many studies to enhance its potential neuroprotective effects, including limiting proliferation, resuming neurogenesis, and improving long-term transplant survival [165].

Cell-replacement therapies for AD are currently being tested been in humans, and the majority of these therapies use MSCs from various sources. Stem cell therapy for AD is not yet in phase 3 clinical trials. Using the findings from the initial trial, the effectiveness, tolerance, and safety of transplanting were evaluated. Allogeneic human umbilical cord MSCs (hUCB-MSCs) were injected into the right precuneus and hippocampus of her patients with mild to moderate AD in a phase 1 clinical experiment carried out in South Korea in 2015. This trial investigated the treatment’s effectiveness and safety. Safety, survival and tolerability goals were met for all primary and secondary endpoints [166]. In addition, a case study using intrathecal injection of autologous MSCs demonstrated significant improvement in clinical symptoms in two patients and overall glucose metabolism in the brain as determined by 18F-fluorodeoxyglucose PET imaging [167]. These effective paradigms imply that MSCs have a minimal risk of side effects and are suitable for widespread usage in upcoming AD clinical trials. According to previous studies [168], Alzheimer’s patients exhibit region-specific basal forebrain cholinergic system depression (BFCS). The utilization of cholinergic cell-based transplantation as a therapeutic strategy might become a reality thanks to advancements in stem cell biology.

According to two investigations, model animals’ cognitive function was greatly enhanced by the transplanting of both human foetal basal forebrain cholinergic cells and human chorion-derived basal forebrain cholinergic progenitor cells [169]. A description of the excitatory and inhibitory imbalance that served as an example of the pathophysiology of Alzheimer’s disease has been provided. This theory implies that the main focus for improving cognitive function in AD patients may be the GABAergic system [170]. Shrestha et al. [171] transplanted human GABAergic interneuron progenitor cells made from hESCs into the hippocampus of rodents and discovered that the transplanted interneurons were better developed and had intricate dendrites. In mice models of neurodegeneration, neurogenic transcription factors or RNA-binding proteins have been shown to transform glial cells into functioning neurons [172]. Moreover, newly generated neurons have the ability to be innervated, repopulated, and ameliorate movement deficits in PD models [173].

In AD mice models, direct reprogramming of astrocytes and neuroglia 2 (NG2) cells results in functioning neurons [174]. In addition, there is proof that microglia can convert into neurons in vivo [175]. ApoE, TREM2, and CD33 have been identified as key genes involved in the intermediate state of disease-associated microglia (DAM, also referred to as microglial neurodegenerative phenotype) by most recent single-cell RNA sequencing studies of microglia from AD-transgenic (Tg) mice [176].

Delivery of cell therapy to the brain has been demonstrated to trigger an immunological response in models of Parkinson’s disease. They demonstrated that using MHS-matched grafts greatly reduced immune responses when compared to using non-MHS-matched grafts, but immunological responses did not seem to be totally avoided. Immune rejection is thus a significant problem in the treatment of AD cells. Major Histocompatibility Complex (MHC) matching has been demonstrated in animal experiments to improve graft survival following organ transplantation [177]. Surprisingly, transplanted cells can be modified in vitro to reduce intracellular immunogenicity using genome editing engineering or used as vectors to enhance the immunological milieu in vivo and can dramatically reduce the risk of immune rejection [178].

When transplanted into non-human primate models, MHC-matched allografts have been found to decrease immune rejection and increase survival [179]. Beta 2-microglobulin (B2M) gene knockout and interference with human leukocytes antigen A (HLA-A) and B (HLA-B) may also lessen the immunogenicity of stored allografts [179].

To evaluate the effectiveness of individualized treatments, disease-in-a-dish models with patient-specific data can be created using patient-derived autologous cells. Genetic alterations or modifications can render transplanted cells resistant or refractory to disease pathologies prior to transplantation. No adverse effects were observed in this area from studies of human immunodeficiency virus (HIV) and acute lymphoblastic leukemia (ALL) [180]. This preventive approach focuses on the preclinical stages of AD, when only a few damaged brain cells need to be repaired. The problem with this method is that the overall medical procedure is expensive and time consuming. Chronic illnesses like Alzheimer’s disease, on the other hand, might not call for the quick synthesis of pre-made cells like other acute illnesses do. Prioritizing this design will boost reprogramming efficiency and safety while lowering expenses [181–183].

## Conclusion

Future multilineage techniques and stem cell models may be able to detect early interactions between genes and molecules and developmental abnormalities in cells before they eventually become dysfunctional and die in AD. Disease models generated from iPSCs possess a level of detail that allows us to determine the neurological underpinnings of disease states and carefully examine the mechanisms behind the development and progression of such diseases. Combining stem cell-derived models improves the accuracy of detecting early immune cell changes and determining their contribution to AD pathogenesis. Future research should improve many issues related to stem cells. A fundamental problem is the immaturity of stem cell-derived cell types, which complicates the handling of these cells after transplantation into patients. By combining transplanted cells with a cell-engineering toolkit that can target endogenous loci or disrupt gene expression at specific loci without altering therapeutic efficacy, transplanted cells can be immunogenic and genetically modified. HLA-matched cell banks are commonly used because gene editing can be used to reduce the immunogenicity of transplanted cells. Cell therapy can now be used for AD clinical research by delivering neurotrophic factors, replacing lost cells, promoting endogenous neurogenesis, modulating inflammatory responses, and altering the host microenvironment. Stem cell therapy combined with precision medicine is probably the most efficient treatment for AD. Using hiPSC-derived models as a predictive platform could accelerate the development of precision medicine and “clinical trials in a dish”, making AD therapeutics more likely to be effective. Currently, there are no proven methods of AD stem cell therapy and it is still in its early stages. Given the many failures of AD treatment trials, we believe that stem cell-based AD treatments will shock us in the near future.

## Data Availability

All data are available in the manuscript.

## Abbreviations

AD: Alzheimer’s disease
iPSCs: Induced pluripotent stem cells
FAD: Familial AD
SAD: Sporadic AD
NFTs: Neurofibrillary tangles
Aβ: Amyloid β
*APP*: Amyloid precursor protein
p-tau: Hyperphosphorylated tau
β-Secretase: Beta-secretase
γ-Secretase: Gamma-secretase
α-Secretase: Alpha-secretase
CAA: Cerebral amyloid angiopathy
CSF: Cerebrospinal fluid
MAPT: Microtubules associated protein
BBB: Blood–brain barrier
*PSEN1*: Presenilins 1
*PSEN2*: Presenilins2
GWAS: Genome-wide association study
*APOE4*: Apolipoprotein E
*TREM2*: Triggering receptor expressed on myeloid cells 2
*ABCA7*: ATP-binding cassette sub-family A member 7
*SORL1*: Sortilin-related receptor, L (*DLR* class
*OCT4*: Octamer-binding transcription factor 4
*SOX2*: Sex determining region Y-box 2
*KLF4*: Kruppel-like factor 4
DS: Down’s syndrome
GSM: γ-Secretase modulator
GSI: γ-Secretase inhibitors
LRP1: Low-density lipoprotein receptor-related protein 1
HiPSCs: Human induced pluripotent stem cells
WT: Wild-type
HPCs: Hematopoietic progenitor cells
PLCG2: 1-Phosphatidylinositol-4,5-bisphosphate phosphodiesterase gamma-2
scRNA-seq: Single-cell RNA-sequencing
ZFN: Zinc finger nuclease
TALENs: Transcription activator-like effector nucleases
CRISPR: Clustered regularly interspaced short palindromic repeats
Cas9: CRISPR-associated protein
crRNA: CRISPR RNA
transRNA: Trans-activating RNA
iBECs: Induced brain endothelial cells
MSCs: Mesenchymal stem cell
hUC-MSCs: Human umbilical cord mesenchymal stem cells
HGF: Hepatocyte growth factor
MenSCs: Menstruation stem cells
BM-MSCs: Bone marrow mesenchymal stem cells
EVs: Extracellular vesicles
OB-NSCs: Human olfactory bulb (OB)-derived NSCs
CNTs: Carbon nanotubes
hESCs: Human embryonic stem cells
MGE: Medial ganglionic eminence
TEPs: Thymic epithelial progenitor cells
IMRC: Immune and matrix regulatory cells
hUCB-MSCs: Human umbilical cord MSCs
BFCS: Basal forebrain cholinergic system depression
*NG2*: Neuroglia 2
*MHC*: Major Histocompatibility Complex
*B2M*: Beta 2-microglobulin
*HLA-A*: Human leukocytes antigen A
*HLA-B*: Human leukocytes antigen B
HIV: Human immunodeficiency virus
ALL: Acute lymphoblastic leukemia
